# Trimming of Imperfect Cylindrical Fused Silica Resonators by Chemical Etching

**DOI:** 10.3390/s19163596

**Published:** 2019-08-18

**Authors:** Yunfeng Tao, Yao Pan, Shilong Jin, Yonglei Jia, Kaiyong Yang, Hui Luo

**Affiliations:** College of Advanced Interdisciplinary Studies, National University of Defense Technology, NO. 47 Yanwachi Road, Kaifu District, Changsha 410073, China

**Keywords:** cylindrical resonator, frequency split, chemical trimming

## Abstract

The cylindrical resonator gyroscope (CRG) is a kind of solid-state gyroscope with a wide application market. The cylindrical resonator is the key component of CRG, whose quality factor and symmetry will directly affect the performance of the gyroscope. Due to the material properties and fabrication limitations, the actual resonator always has some defects. Therefore, frequency trimming, i.e., altering the local mass or stiffness distribution by certain methods, is needed to improve the overall symmetry of the resonator. In this paper, we made further derivation based on the chemical trimming theory proposed by Basarab et al. We built up the relation between the frequency split and the balanced mass to determine the mass to be removed. Chemical trimming experiments were conducted on three cylindrical fused silica resonators. The frequency splits of the three resonators were around 0.05 Hz after chemical trimming. The relation between frequency split and balanced mass established from experimental data was consistent with the theoretical calculation. Therefore, frequency split can be reduced to lower than 0.05 Hz under rigorous theoretical calculation and optimized chemical trimming parameters.

## 1. Introduction

Axisymmetrical vibratory gyroscopes have gained great attention among researchers for their wide applications in aircraft, land vehicles, ships, individual devices [1,2,3,4,5], etc. They are a kind of Coriolis vibratory gyroscope which uses the angular or axial displacement of vibration modes in the thin shell to detect rotation. The cylindrical resonator gyroscope is newly developing, with great potential of high accuracy, small size, and considerable reliability [6,7,8,9]. An ideal resonator has two spatially orthogonal vibration modes with the same natural frequency and indeterminate circumferential location [10]. However, the presence of imperfections will severely destroy the symmetry of the resonator, causing the two equal natural frequencies to split into two. Even a small frequency split will cause a significant decline in the performance of the gyroscope. Therefore, the frequency split must be decreased by special procedures known as frequency trimming [11].

Methods to reduce the frequency split can be classified into two categories. One is altering the local stiffness temporarily, usually by electrostatic force, and the other is changing the local mass and stiffness by permanently removing or adding a volume of mass. Electrostatic trimming selectively changes the stiffness of the resonator by applying electrostatic force to the specific position, which does not destroy the structure of the resonator and is always used in real-time online mode matching. However, electrostatic trimming requires additional voltages, which increase energy consumption and the complexity of the system, causing additional noise [12]. In the meantime, due to the limitation of voltages applied to the electrodes, the range of electrostatic trimming on frequency split is small. Gallacher et al. established a mathematical model of electrostatic trimming, which allowed the determination of appropriate trim electrodes and voltages [13]. Gallacher then improved the model and adopted a dynamic trimming algorithm that can monitor the frequency split in real time, successfully reducing the frequency split from 1.5 Hz to 6 mHz, realizing high-accuracy electrostatic trimming [14]. Xiao et al. adjusted the local stiffness of the disk resonator by the electrostatic force to reduce frequency split. Each of the trimming electrodes was equivalent to two rows of radial springs having a negative stiffness. On this basis, the relationship between equivalent stiffness, each trimming electrode, and the frequency split was obtained [15]. Moreover, electrostatic trimming is suitable for the trimming of microelectromechanical system (MEMS) resonators that is difficult to remove or add material, which enables MEMS resonators to acquire a performance better than a few degrees per hour [16].

Various methods can be used to add or remove mass from the resonator permanently. Ion beam deposition is widely applied to adding a small amount of mass to MEMS resonators. Chang et al. presented a frequency tuning technique on cantilever-shape nano-resonators using focused ion beam-chemical vapor deposition [17]. Enderling et al. deposited Pt on a polysilicon cantilever resonator within a surface area of 13 um × 5 um. The natural frequency was changed after deposition. However, the Q factor decreased significantly due to thermoelastic damping [18]. Courcimault et al. also reported a large Q-decrease, from 25,000 to 5,000, after the metal deposition onto the top surface of micro resonator [19]. Therefore, removing mass from resonators is preferred to avoid the severe decrease of the Q factor.

Mechanical trimming was reported to remove mass from resonators, especially those made of metal materials. Wang et al. reported a technique on permanent frequency split reduction of fused quartz micro wineglass resonators by directional lapping [20,21]. Raspopov et al. presented a method of manually removing mass from the metal resonator [22]. Yi et al. analyzed the influence of different trimming positions on the eigenfrequency of the resonator using the equivalent mass-defect model, and the frequency split was reduced to 0.028 Hz by mechanical trimming [23]. Femtosecond laser ablation, with advantages of small thermal diffusion depth, high-precision, and low damage, is used in the frequency tuning of a resonator. Zeng et al. carried out simulation and experimental verification on the top trimming of the cylindrical resonator, and the frequency split was decreased to 0.06 Hz by femtosecond laser trimming [24]. Lu et al. analyzed the causes of the frequency split of a micro shell resonator with T-shaped masses by theory and finite element simulation, and the T-shaped micro-resonator was trimmed by a femtosecond laser with a final frequency split of 0.9 Hz [25].

Chemical etching [26,27] is another possible trimming method of fused silica resonator proposed by Basarab et al. Following this approach, Wang et al. verified that removing mass at low-frequency axis can effectively reduce frequency split by simulation. In the experiment, they selected appropriate inclined angle and immersion depth, combined with the actual chemical trimming conditions and the structure of the hemispherical resonator, successfully reducing the frequency split of the hemispherical fused silica resonator to 0.025 Hz [28].

In this paper, the principle of chemical trimming was further developed. In Section 2, the relationship between the frequency split and the balanced mass was deduced, revealing a linear variation of frequency split with the total balanced mass by chemical trimming. This result can be used to predict the balancing mass according to the current frequency split. Chemical trimming experiments were conducted on three cylindrical fused silica resonators. The experiment was presented in Section 3, and a fairly good result was achieved with a final frequency split around 0.05 Hz after several rounds of chemical trimming. The relation between frequency split and the balanced mass was consistent with the theoretical calculation. In Section 4, the possible errors and the prominent advantages of the chemical trimming methods were discussed.

## 2. Chemical Trimming Principle

Chemical trimming utilizes a suitable chemical solution to remove unbalanced mass at specific locations of the resonator. The basic process of chemical trimming is immersing the edge of the cylindrical resonator into the etching solution at an appropriate inclined angle and depth. This process can change the local mass and stiffness distribution, which can decrease the frequency split effectively. To eliminate the frequency split controllably, we need to establish the relation between the etched mass and the frequency split.

**Figure 1:**
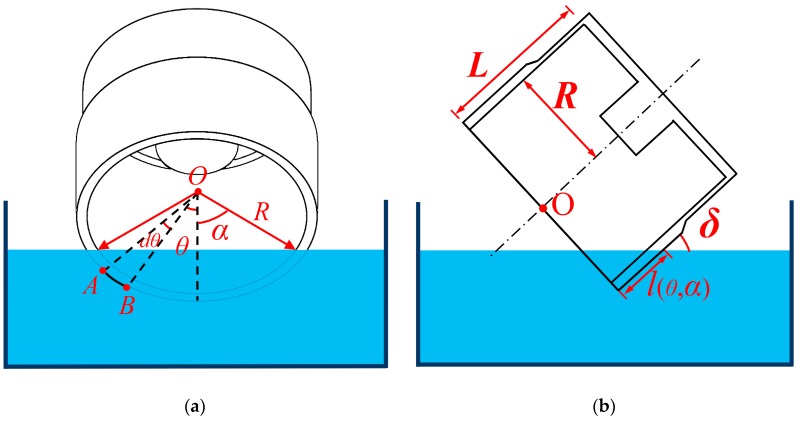
The schematic of chemical trimming: (**a**) The front view of the etching geometry; (**b**) The half-section view of the etching geometry.

The schematic of chemical trimming is shown in Figure 1, where *R* is the radius of the cylindrical resonator, *δ* is the inclined angle, *L* is the axial length of the resonator, 2*α* is the central angle of the wedge-shaped part immersed in the etching solution, *θ* is the circumferential angle, *dθ* is the differential element at the circumferential angle *θ*, and *l*(*θ*, *α*) is the length along the axial direction among the immersed part at the circumferential angle *θ*. The cylindrical resonator was partly immersed in the etching solution with the inclined angle *δ* and the zenith angle α.

A portion of the mass is removed from the edge of the cylindrical resonator during each etching. Due to the geometrical distribution of the removed mass, the coefficients of the first four harmonics will vary with the circumferential angle accordingly. Therefore, we need to figure out the relationship between the inclined angle, immersed depth, and the mass etched. For the sake of convenience, we set the circumferential angle *θ* = 0° at the lowest point of the immersed part. According to the geometry relationship depicted in Figure 1, we assume the maximum value of *l*(*θ*, *α*) remained constant. Hence, the depth at an arbitrary circumferential angle is [27]

(1) $$l\left(\theta ,\alpha \right)=l_{max}\frac{\cos\;\theta -\cos\;\alpha }{1-\cos\;\alpha },\;\left|\theta \right|<\alpha$$

where $l_{max}=\frac{R\left(1-\cos\;a\right)}{\tan\;\delta }$ is the maximum value of *l*(*θ*, *α*).

Expand *l*(*θ*, *α*) into a Fourier series with respect to *α* [27]

(2) $$l\left(\theta ,\alpha \right)=\frac{l_{max}}{\pi \left(1-\cos\;\alpha \right)}\left[\left(\sin\alpha -\alpha \cos\alpha \right)+\left(\alpha -\sin\alpha \cos\alpha \right)\cos\theta +2\sum_{k=2}^{\infty }\frac{k\cos k\alpha \sin\alpha -\cos\alpha \sin k\alpha }{k\left(1-k^{2}\right)}\cos k\theta \right]$$

The first four harmonics are the main error sources which deteriorate the performance of CRG. Therefore, we only take the first four harmonics into account [27]

(3) $$l\left(\theta ,\alpha \right)=l_{max}\left[F_{0}+F_{1}\cos\theta +F_{2}\cos2\theta +F_{3}\cos3\theta +F_{4}\cos4\theta \right]$$

It is noteworthy that the coefficients of the first four harmonics of the resonator changed after each etching. As shown in Figure 1, the arc length $\overset{⌢}{\mathrm{AB}}$ at the circumferential angle *θ* is *Rdθ*. Therefore, the area element at the circumferential angle *θ* is *l(θ*, *α*) *Rdθ*. Performing integral of *l*(*θ*, *α*) *Rdθ* on angle *θ*, we can obtain the area of each immersion with parameters *α* and *δ*. Multiplying the area by the etching thickness *h* gives the volume of chemical trimming, assuming that *h* is a constant value which is independent of *α* and *δ*. Therefore, the mass removed in each immersion can be expressed as

(4) $$M=2\rho hR\int_{0}^{\alpha }l\left(\theta ,\alpha \right)d\theta$$

where *ρ* is the density of the cylindrical resonator, *h* is the total thickness etched of the immersed part.

Because the balance of the *k*th harmonic error will introduce *mk*th (*m* = 2, 3, 4…) harmonic defects, a complete trimming process needs to balance the first four harmonics in turn. It is quite complex to remove all the four harmonic errors thoroughly. At the current stage, we only pay attention to the trimming of the fourth harmonic error.

The removing rate of the fourth harmonic error reaches the maximum when *F*_4_(*α*) is at its maximum value. Hence, appropriate angle *α* is needed to acquire the maximum removing rate. Perform derivation of the *F*_4_(*α*) on angle *α* [27]

(5) $$F_{4}^{\prime }\left(\alpha \right)=\frac{1}{15}\left(\cos\alpha +4\cos2\alpha +9\cos3\alpha +6\cos4\alpha \right)$$

When *F*_4_(*α*) = 0, the angle *α* = 0.521 rad and *F*_4_(*α*) reaches the maximum value 0.1386.

During the practical trimming process, the unbalance mass of the fourth harmonic error is removed along the error orientation by four times of etching at four equidistributed positions. Therefore, Equation (3) can be written as [27]

(6) $$\begin{array}{ll} l_{4}\left(\theta ,\alpha \right) & =l_{max}\sum_{0}^{4}F_{k}\left(\alpha \right)\sum_{j=0}^{3}\cos k\left(\theta +j\frac{\pi }{2}\right)\\ & =4l_{max}\left[F_{0}\left(\alpha \right)+F_{4}\left(\alpha \right)\cos4\alpha \right] \end{array}$$

Equation (6) demonstrates that the trimming on the fourth harmonic error does not affect the first three. Substituting Equation (6) into Equation (4), the total mass removed after four times of etching is

(7) $$M_{T4}=M_{0}+M_{4}=2\rho hRl_{max}\left[4F_{0}\left(\alpha \right)\alpha +F_{4}\left(\alpha \right)\sin4\alpha \right]$$

where *M*_0_ is the mass removed uniformly by chemical trimming and *M*_4_ is the fourth harmonic mass removed by chemical trimming.

**Figure 2:**
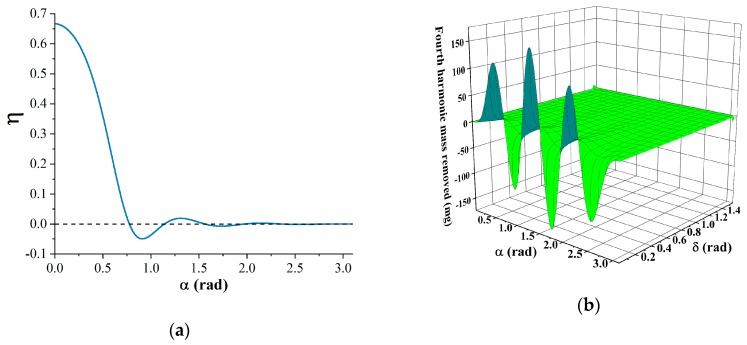
The relation between the fourth harmonic mass removed by chemical trimming and the etching parameters: (**a**) Dependence of the trimming efficiency on angle *α*; (**b**) Dependence of the fourth harmonic mass removed by chemical trimming on angle *α* and *δ*.

Defining trimming efficiency as *η* = *M*_4_/(*M*_0_ *+ M*_4_), the dependence of the trimming efficiency on angle α is shown in Figure 2a. Larger trimming efficiency means less total mass needs to be removed for the same frequency split. The trimming efficiency drops sharply with the growth of angle α. Assuming the etching thickness *h* = 0.1 mm, the dependence of the fourth harmonic mass removed by chemical trimming on angle α and *δ* is shown in Figure 2b. The fourth harmonic mass removed by chemical trimming fluctuates with the growth of angle α. The absolute value of *M*_4_ increases rapidly with the decrease of angle *δ*. Trimming with the parameters in the area where *M*_4_ is negative will increase the total fourth harmonic of the resonator mass distribution, which will result in the increase of frequency split. The selection of angle α should take both the trimming efficiency and the operability into consideration. Smaller angle α would result in more difficulty in the precise control of depth during experiments. The selection of the angle *δ* depends on the current frequency split. Smaller angle *δ* is preferred when the frequency split gets lower.

According to the theoretical derivation presented in Appendix A, the frequency split is proportional to the fourth harmonic error of cylindrical resonators. Chemical trimming decreased frequency split by removing the fourth harmonic error of the resonator. Therefore, we assumed that the fourth harmonic of the resonator mass distribution is equal to the fourth harmonic mass removed by chemical trimming

(8) $$\Delta m_{4}=\varepsilon_{4}\frac{m}{f}\Delta f=2\rho hRl_{max}F_{4}\left(\alpha \right)\sin4\alpha$$

where Δ*f* is the frequency split of the resonator, *f* is the natural frequency of the perfect resonator, *m* is mass of the resonator, Δ*m*_4_ is the fourth harmonic of the resonator mass distribution, and *ε*_4_ is a coefficient which depends on the structure of the resonator.

Therefore, the total mass removed is a linear function of the frequency split

(9) $$M_{T4}=2\rho hRl_{\max}4F_{0}\left(\alpha \right)\alpha +\varepsilon_{4}\frac{m}{f}\Delta f$$

For our cylindrical resonator, we set *l_max_* = 1.25 mm, *δ* = 53°. Substituting the angle *α* = 0.521 rad into Equation (7), the total area of the immersed parts is 10.94 mm^2^. Substituting the structure parameters into Equation (9), the total mass to be removed with frequency split Δ*f* is

(10) $$M_{T4}=15.7896h+0.5836\varepsilon_{4}\Delta f(mg)$$

## 3. Experiment and Results

**Figure 3:**
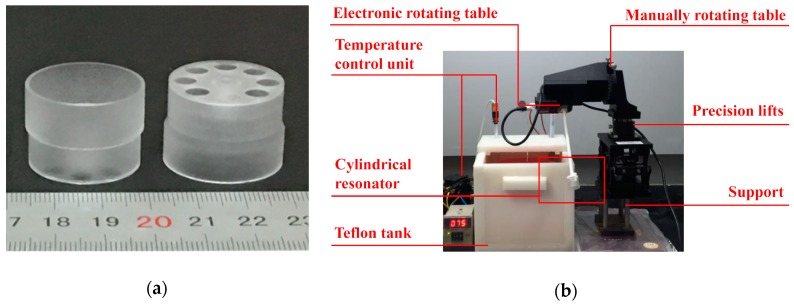
The cylindrical resonator and the experimental setup: (**a**) Cylindrical fused silica resonator; (**b**) Experimental setup of the chemical trimming.

A 25 mm-diameter cylindrical fused silica resonator with eight holes on the bottom and a stem inside the shell is shown in Figure 3. The size and location of the eight holes were elaborately designed to achieve a desired effective stiffness of the bottom plate. The structure and design considerations of the resonator was demonstrated in our previous paper [7] by Pan et al.

For accurate chemical trimming, it is of great importance to control the immersed depth *l_max_* and the inclined angle *δ* precisely during the trimming process. We designed a special chemical trimming system to achieve this goal. The cylindrical resonator is mounted on a Teflon fixture, the immersed depth *l_max_* is controlled by a precision lift, and the inclined angle *δ* is adjusted by the manually rotating table with an angular resolution of 5′. The etching position is controlled by the electronic rotating table.

First, the orientation of the fourth harmonic error and the frequency split were determined with the assist of the laser Doppler vibrometer (PSV-500, Polytec) following the method proposed by Wang et al. [28]. Second, the location found in the first step was set as the initial etching position. The etching time *t* was determined by the measured etching rate (mass removed per unit time by the etching solution) and the current frequency split. Starting from the first etching position, we immersed the resonator into the etching solution with the calculated depth *l_max_* and inclined angle *δ*. The electronic rotating table rotated 90° for the next etching position, and the etching was performed four times in a single chemical trimming procedure. After chemical trimming, the mass of the resonator was measured by a 10 ug-precision balance (AUW120D, SHIMADZU).

**Figure 4:**
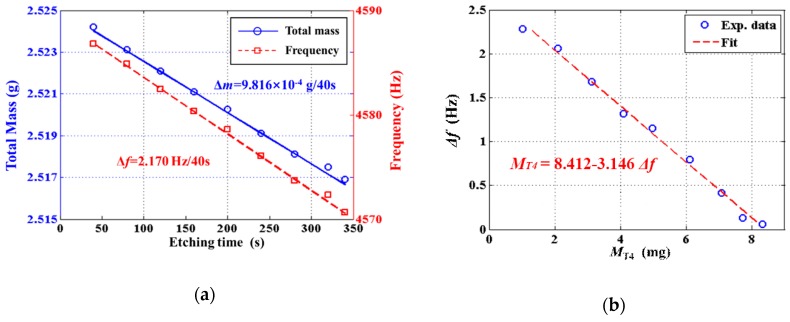
The chemical trimming results on resonator #I01: (**a**) Variations of the total mass and the lower natural frequency; (**b**) Variation of the frequency split Δ*f* on the total balancing mass *M_T_*_4_.

The cylindrical resonator numbered #I01 was chemically trimmed nine times. As shown in Figure 4a, the lower natural frequency and the resonator mass decreased linearly with etching time. The variation of the total mass and lower natural frequency for an etching period of 40 s was about 0.9816 mg and 2.17 Hz, respectively. The total etching thickness *h* was about 0.373 mm according to the immersed area and the total mass removed.

Figure 4b revealed a linear variation of frequency split Δ*f* with the total balancing mass *M_T_*_4_, the numerical relationship between which was

(11) $$M_{T4}=8.412-3.146\Delta f$$

**Figure 5:**
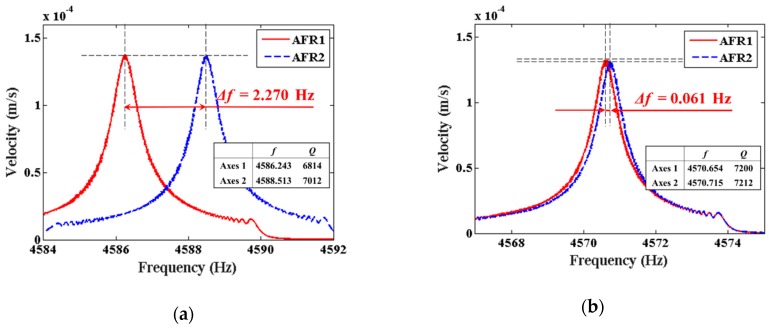
The variations of frequency split and Q factor of resonator #I01: (**a**) Before chemical trimming; (**b**) After chemical trimming.

The frequency split and Q factor of the resonator before and after chemical trimming are shown in Figure 5. The lower natural frequency decreased from 4586.243 Hz to 4570.654 Hz after nine rounds of trimming, while the higher natural frequency decreased from 4588.513 Hz to 4570.715 Hz. As a result, the frequency split decreased from 2.270 Hz to 0.061 Hz. Meanwhile, the Q factor of the lower frequency axis increased from 6814 to 7200, and the Q factor of the higher frequency axis increased from 7012 to 7212, as shown in the embedded table in Figure 5a,b. Defining Q factor asymmetry as *δQ* = 2|*Q*_2_ − *Q*_1_|/(*Q*_2_ + *Q*_1_), the Q factor asymmetry achieved an improvement of 2.7%. The improvement of the Q factor and its asymmetry is the most prominent advantage of chemical trimming compared with laser ablation or mechanical treatment.

**Figure 6:**
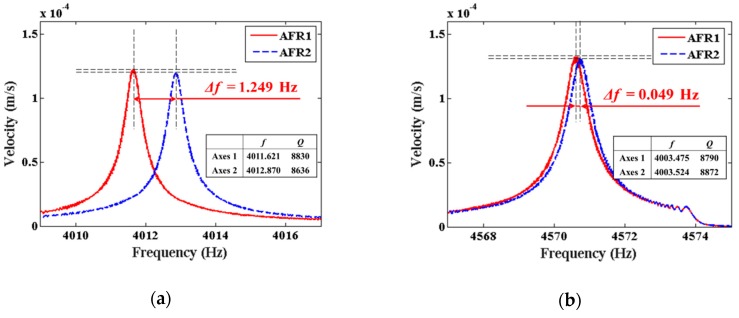

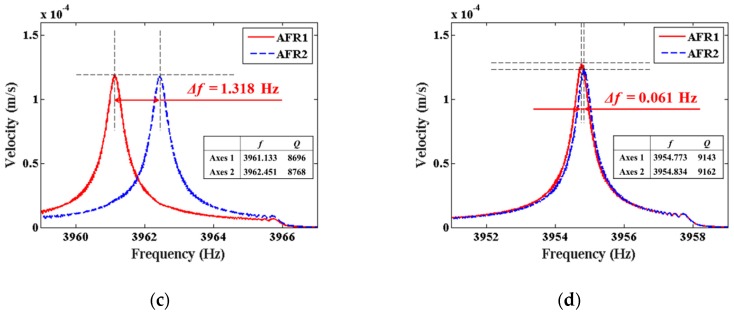
The variation of frequency split and Q factor of #I02 and #I03: (**a**) #I02 before trimming; (**b**) #I02 after trimming; (**c**) #I03 before trimming; (**d**) #I03 after trimming.

Based on the trimming experiments on resonator #I01, chemical trimming was conducted on two other resonators, #I02 and #I03. The frequency splits and Q factors of resonators #I02 and #I03 before and after trimming are shown in Figure 6. The frequency split of resonator #I02 decreased from 1.249 Hz to 0.049 Hz after three times of chemical trimming, while the frequency split of resonator #I03 decreased from 1.318 Hz to 0.061 Hz after six times of chemical trimming.

Figure 6 also illustrated that the reduction of frequency split was accompanied by the improvement of Q factor and Q symmetry. Chemical trimming results of three resonators were listed in Table 1, where *f*_1_ and *Q*_1_ stand for the frequency and Q factor of the low-frequency axis, *f*_2_ and *Q*_2_ stand for the frequency and Q factor of the high-frequency axis (not shown in Table 1), the frequency split is Δ*f* = *f*_2_ − *f*_1_, and the Q factor asymmetry is *δQ* = 2|*Q*_2_ − *Q*_1_|/(*Q*_2_ + *Q*_1_). Results showed that by careful control of the etching parameters, frequency tuning by chemical etching is not only feasible, but also has unique advantages on the improvement of Q factors and Q symmetry.

Experimental results demonstrated that the frequency split of cylindrical resonators could be decreased to below 0.1 Hz by several rounds of chemical trimming. However, the average variation of mass, natural frequency, and frequency split are highly resonator-dependent. Therefore, to remove mass quantitatively, the calibration of etching rate is needed for cylindrical resonators with different structures at this stage.

## 4. Discussions

Taking the total thickness removed of resonator #I01 into Equation (10), the intercept of is 5.894. Compared with the intercept of Equation (11), there is a difference of 29.9%. This difference is mainly caused by the following two reasons. One is that approximations were adopted in the theoretical derivation. During the theoretical derivation, the geometry of the resonator was greatly simplified into a cylindrical shell. However, the structure of the resonator used in the experiments was much more complicated, which has a stepped structure of the cylindrical shell and a bottom plate with eight equidistributed holes. Moreover, the thickness and the stiffness variation caused by chemical trimming were not considered in this model. Approximations of the theoretical calculation are at the expense of precision. The other is that it is difficult to control the chemical etching conditions precisely because of the instability of the etching solution and the errors from manual operation. Therefore, the specific numerical relationship between the frequency split and the fourth harmonic error is experimentally calibrated at this stage. For the further decrease of frequency split, efforts on high-precision position control and higher-accuracy automated operation are needed. We are exploring the possibility of improving the chemical trimming procedure, so that by elaborate theoretical calculation and careful control of trimming parameters, the frequency split can be reduced to below 0.1 Hz with one-time chemical trimming.

Results from three cylindrical fused silica resonators also showed that the Q factors all improved slightly after chemical trimming. This may be attributed to the removing of the hydrate and damaged surface layer [29,30], as indicated by Lunin et al. As other trimming methods usually decrease the Q factor of resonators, the improvement of Q factor and Q symmetry is the most prominent advantage of chemical trimming. However, to investigate the degree of Q factor improvement, measurement in vacuum conditions needs to be performed.

## 5. Conclusions

In summary, following the method proposed by Basarab et al., this paper reported experimental methods and results of chemical trimming on cylindrical fused silica resonators. The theoretical aspects of the chemical trimming were briefly analyzed, and by theoretical calculation, the key parameters of chemical trimming were analyzed and selected according to the trimming efficiency of the fourth harmonic error. Experiments were performed on three cylindrical fused silica resonators. The frequency splits of the three resonators were trimmed to lower than 0.1 Hz after several rounds of chemical trimming, and the frequency split decreased linearly with the increase of the total mass removed, demonstrating the feasibility of the chemical trimming method. Moreover, the Q factor and Q symmetry improved slightly after chemical trimming, which is a prominent advantage of the chemical trimming method. Numerical comparisons between the theoretical model and experimental results were demonstrated, and error sources were discussed. As chemical trimming has the advantage of low cost, simple operation, high efficiency, and Q-factor preservation, with high-precision position control and higher-accuracy automated operation, chemical trimming can be a practical medium-accuracy method for the trimming of fused silica resonators.

## Figures and Tables

**Table 1:** Frequency trimming results of three cylindrical fused silica resonators.

Resonator	Before Etching				After Etching			
	*f*_1_ (Hz)	Δ*f* (Hz)	*Q*_1_	*δQ* (%)	*f*_1_ (Hz)	Δ*f* (Hz)	*Q*_1_	*δQ* (%)
#I01	4586.243	2.270	6814	2.864	4570.654	0.061	7200	0.167
#I02	4011.621	1.249	8830	2.221	4003.475	0.049	8790	0.929
#I03	3961.133	1.318	8696	0.825	3954.773	0.061	9143	0.207

**Figure A1:**
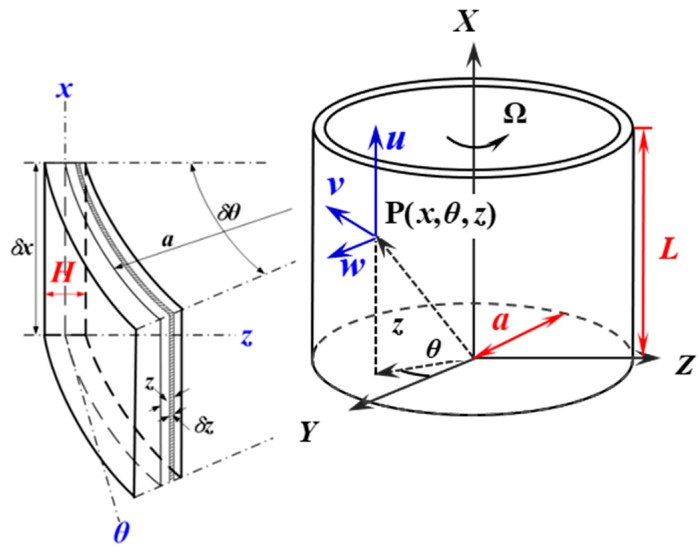
Coordinate system and geometry of the cylindrical shell.

## Appendix A

The theoretical relationship between frequency split and the fourth harmonic error was deduced as follows:

The geometry of the cylindrical shell and the definition of the coordinate system are shown in Figure A1 where *L* represents the height, *a* represents the mid-surface radius and *H* represents the thickness of the cylindrical shell. Let the orthogonal coordinate system (*x*, *θ*, *z*) of the shell coincides with the axial, tangential and radial directions of the middle surface, respectively, and (*i*, *j*, *k*) is the unit vector of the (*x*, *θ*, *z*) system in three directions, respectively.

The displacement of a point P on the surface of the cylindrical shell is represented by (*u*, *v*, *w*) at (*x*, *θ*, *z*). The eigenfunctions of the displacement of the *n* = 2 mode for a cylindrical shell usually can be expressed by two generalized coordinates (*C*, *S*) as [31,32]

(A1) $$\begin{array}{l} u\left(x,\theta ,t\right)=U\left(x\right)\left[C\left(t\right)\cos2\theta +S\left(t\right)\sin2\theta \right]\\ v\left(x,\theta ,t\right)=V\left(x\right)\left[C\left(t\right)\sin2\theta -S\left(t\right)\cos2\theta \right]\\ w\left(x,\theta ,t\right)=W\left(x\right)\left[C\left(t\right)\cos2\theta +S\left(t\right)\sin2\theta \right] \end{array}$$

where *U*(*x*), *V*(*x*), *W*(*x*) are the eigenfunctions of the system.

The Lagrangian of the *n* = 2 mode is given by

(A2) $$L\left(C,S,\dot{C},\dot{S}\right)=T_{c}-U_{c}$$

where *T_c_* and *U_c_* represent the kinetic energy and potential energy, respectively. Substituting *T_c_* and *U_c_* into the Lagrangian function

(A3) $$\begin{array}{l} \frac{d}{dt}\left(\frac{\partial L}{\partial \dot{C}}\right)-\frac{\partial L}{\partial C}=0\\ \frac{d}{dt}\left(\frac{\partial L}{\partial \dot{S}}\right)-\frac{\partial L}{\partial S}=0 \end{array}$$

To obtain the kinetic equation of the cylindrical shell from Equation (A3), we need to deduce the specific expressions of *T_c_* and *U_c_*. Consider a point P (*x*, *θ*, *z*); the position vector can be written as

(A4) R=ui+vj+wk

Suppose the direction of the external angular velocity coincides with the axial direction of *X*

(A5) Ω=Ωi

Therefore, the velocity vector of P in the inertial space is

(A6) $$V=\dot{R}+\Omega \times R$$

The kinetic energy of the cylindrical resonator is

(A7) $$\begin{array}{ll} T_{c}= & \frac{\rho \mathrm{aH}}{2}\int_{0}^{2\pi }\int_{0}^{L}\left(\vec{V}\times \vec{V}\right)\mathrm{dxd}\theta \\ & =\frac{\rho \mathrm{aH}}{2}\int_{0}^{2\pi }\int_{0}^{L}\left[{\dot{u}}^{2}+{\dot{v}}^{2}+{\dot{w}}^{2}+2\Omega \left(\dot{w}v-\dot{v}w\right)+\Omega^{2}\left(v^{2}+w^{2}\right)\right]\mathrm{dxd}\theta \end{array}$$

The frequency of the vibration mode of the cylindrical shell is much bigger than the external angular velocity, then an assumption as Ω_2_ << ω_2_ is reasonable to apply this model. Equation (A7) can be simplified into

(A8) $$\begin{array}{ll} T_{c} & =\frac{\rho aH}{2}\int_{0}^{2\pi }\int_{0}^{L}\left[{\dot{u}}^{2}+{\dot{v}}^{2}+{\dot{w}}^{2}+2\Omega \left(\dot{w}v-\dot{v}w\right)\right]dxd\theta \\ & =T_{c0}+T_{c\Omega } \end{array}$$

where *T_c_*_0_ and *T*_*c*Ω_ represent the vibrational kinetic energy and the kinetic energy associated with external angular velocity, respectively.

(A9) $$\begin{array}{l} T_{c0}=\frac{\rho aH}{2}\int_{0}^{2\pi }\int_{0}^{L}\left({\dot{u}}^{2}+{\dot{v}}^{2}+{\dot{w}}^{2}\right)dxd\theta \\ T_{c\Omega }=\frac{\rho aH}{2}\int_{0}^{2\pi }\int_{0}^{L}\left[2\Omega \left(\dot{w}v-\dot{v}w\right)\right]dxd\theta \end{array}$$

Substituting Equation (A1) into Equation (A9)

(A10) $$\begin{array}{l} T_{c0}=\frac{1}{2}I_{0}\left({\dot{C}}^{2}+{\dot{S}}^{2}\right)\\ T_{c\Omega }=\frac{1}{2}\cdot 2\Omega I_{\Omega }\left(C\dot{S}-S\dot{C}\right) \end{array}$$

where $I_{0}=\pi \rho aH\int_{0}^{L}\left(U^{2}+V^{2}+W^{2}\right)dx$ and $I_{\Omega }=\pi \rho aH\int_{0}^{L}\left(2VW\right)dx$.

For simplicity, let the initial circumferential angle of the fourth harmonic error *θ*_4_ = 0. Therefore, Equation (A8) can be written as

(A11) $$\begin{array}{ll} T_{c}^{\prime } & =\frac{aH}{2}\int_{0}^{2\pi }\int_{0}^{L}\left\{\left(\rho_{0}+\rho_{4}\cos4\theta \right)\cdot \left[{\dot{u}}^{2}+{\dot{v}}^{2}+{\dot{w}}^{2}+2\Omega \left(\dot{w}v-\dot{v}w\right)\right]\right\}dxd\theta \\ & =T_{c}+\Delta T_{c} \end{array}$$

where

(A12) $$\Delta T_{c}=\frac{aH}{2}\int_{0}^{2\pi }\int_{0}^{L}\left\{\rho_{4}\cos4\theta \cdot \left[{\dot{u}}^{2}+{\dot{v}}^{2}+{\dot{w}}^{2}+2\Omega \left(\dot{w}v-\dot{v}w\right)\right]\right\}dxd\theta$$

Similarly, Equation (A1) can be written as

(A13) $$\begin{array}{l} {\dot{u}}^{2}=\frac{1}{2}U{\left(x\right)}^{2}\left[{\dot{C}}^{2}\left(1+\cos4\theta \right)+\dot{S}\left(1-\cos4\theta \right)+2\dot{C}\dot{S}\sin4\theta \right]\\ {\dot{v}}^{2}=\frac{1}{2}V{\left(x\right)}^{2}\left[{\dot{C}}^{2}\left(1-\cos4\theta \right)+\dot{S}\left(1+\cos4\theta \right)-2\dot{C}\dot{S}\sin4\theta \right]\\ {\dot{w}}^{2}=\frac{1}{2}W{\left(x\right)}^{2}\left[{\dot{C}}^{2}\left(1+\cos4\theta \right)+\dot{S}\left(1-\cos4\theta \right)+2\dot{C}\dot{S}\sin4\theta \right] \end{array}$$

Due to the orthogonality of trigonometric functions, only the fourth harmonic error has a significant impact on the kinetic energy of the *n* = 2 mode. Evaluate integral of Equation (A12) on the circumferential angle under the assumption that *ρ*_4_/*ρ*_0_ << 1

(A14) $$\begin{array}{ll} \Delta T_{c} & =\frac{1}{4}\rho_{4}aH\left({\dot{C}}^{2}-{\dot{S}}^{2}\right)\int_{0}^{L}\left[U{\left(x\right)}^{2}-V{\left(x\right)}^{2}+W{\left(x\right)}^{2}\right]dx\\ & =\pi I_{E}\left({\dot{C}}^{2}-{\dot{S}}^{2}\right) \end{array}$$

where $I_{E}=\frac{1}{4}\rho_{4}aH\int_{0}^{L}\left(U^{2}-V^{2}+W^{2}\right)dx$.

The vibrational eigenfunctions of the shell remain constant with the assumption that *ρ*_4_/*ρ*_0_ << 1. Therefore, the coefficient of *I_E_* is only determined by the fourth harmonic error. The Lagrangian of the cylindrical shell system can be rewritten as

(A15) $$\begin{array}{ll} L\left(C,S,\dot{C},\dot{S}\right) & =T_{c}^{\prime }-U_{c}\\ & =\pi \left[\begin{array}{r} I_{0}\left({\dot{C}}^{2}+{\dot{S}}^{2}\right)+2\Omega I_{\Omega }\left(C\dot{S}-S\dot{C}\right)+I_{E}\left({\dot{C}}^{2}-{\dot{S}}^{2}\right)\\ -I_{U}\left(C^{2}+S^{2}\right) \end{array}\right] \end{array}$$

Substituting Equation (A15) into Lagrangian, the kinetic equation is obtained as

(A16) $$\begin{array}{l} \ddot{C}-2\eta_{1}\Omega \dot{S}+\omega_{1}^{2}C=0\\ \ddot{S}+2\eta_{2}\Omega \dot{C}+\omega_{2}^{2}S=0 \end{array}$$

where

(A17) $$\begin{array}{l} \eta_{1}=\frac{I_{\Omega }}{I_{0}+I_{E}},\;\omega_{1}=\sqrt{\frac{I_{U}}{I_{0}+I_{E}}}\\ \eta_{2}=\frac{I_{\Omega }}{I_{0}-I_{E}},\;\omega_{2}=\sqrt{\frac{I_{U}}{I_{0}-I_{E}}} \end{array}$$

Take the first-order Taylor expansion of ω_1_ and ω_2_

(A18) $$\begin{array}{l} \omega_{1}=\sqrt{\frac{I_{U}}{I_{0}}}{\left(1+\frac{I_{E}}{I_{0}}\right)}^{-\frac{1}{2}}\approx \sqrt{\frac{I_{U}}{I_{0}}}\left(1-\frac{1}{2}\frac{I_{E}}{I_{0}}\right)\\ \omega_{2}=\sqrt{\frac{I_{U}}{I_{0}}}{\left(1-\frac{I_{E}}{I_{0}}\right)}^{-\frac{1}{2}}\approx \sqrt{\frac{I_{U}}{I_{0}}}\left(1+\frac{1}{2}\frac{I_{E}}{I_{0}}\right) \end{array}$$

Therefore, the frequency split caused by the fourth harmonic error is

(A19) $$\Delta f=\frac{1}{2\pi }\left(\omega_{2}-\omega_{1}\right)=f\frac{I_{E}}{I_{0}}=f\frac{\rho_{4}}{4\pi \rho_{0}}\frac{\int_{0}^{L}\left(U^{2}-V^{2}+W^{2}\right)dx}{\int_{0}^{L}\left(U^{2}+V^{2}+W^{2}\right)dx}$$

where $f=\frac{1}{2\pi }\sqrt{\frac{I_{U}}{I_{0}}}$ is the natural frequency of the perfect resonator.

Therefore, the frequency split is approximately proportional to the fourth harmonic error.

(A20) $$\frac{\Delta f}{f}\propto \frac{\rho_{4}}{\rho_{0}}$$
